# Supplementary figures and images for: Effect of Oxygen Plasma Pre-Treatment on the Surface Properties of Si-Modified Cotton Membranes for Oil/Water Separations
Source: Materials (Basel). 2022 Nov 30;15(23):8551. doi: 10.3390/ma15238551 (PMC9739082; doi:10.3390/ma15238551)

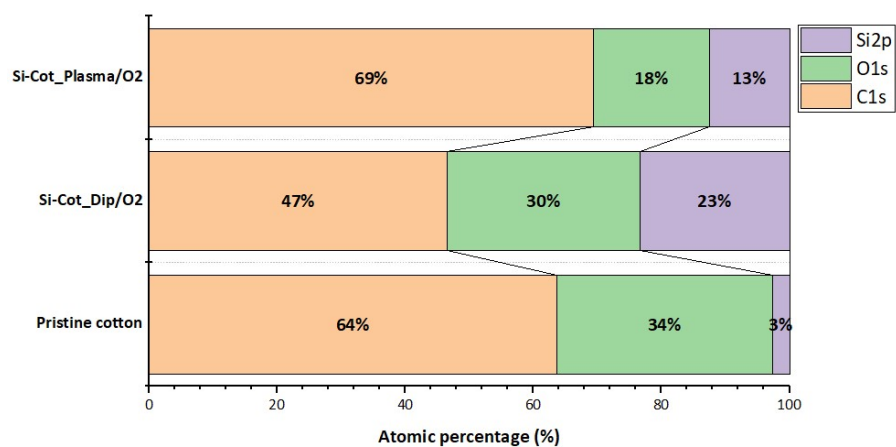

Figure S1. Atomic percentage for Pristine, Si-Cot\_Dip/O<sub>2</sub>, Si-Cot\_Plasma/O<sub>2</sub> samples.

Supplement: Supplementary file 1 [file materials-15-08551-s001.zip › materials-2008052-supplementary.pdf]
